# Spatial-temporal analysis of non-Hodgkin lymphoma in the NCI-SEER NHL case-control study

**DOI:** 10.1186/1476-069X-10-63

**Published:** 2011-06-30

**Authors:** David C Wheeler, Anneclaire J De Roos, James R Cerhan, Lindsay M Morton, Richard Severson, Wendy Cozen, Mary H Ward

**Affiliations:** 1 Occupational and Environmental Epidemiology Branch, Division of Cancer Epidemiology and Genetics, National Cancer Institute (NCI), National Institutes of Health (NIH), Department of Health and Human Services (DHHS), Bethesda, MD, USA; 2 Fred Hutchinson Cancer Research Center and University of Washington, Seattle, WA; 3 Mayo Clinic College of Medicine, Rochester, MN, USA; 4 Radiation Epidemiology Branch, Division of Cancer Epidemiology and Genetics, NCI, NIH, DHHS, Bethesda, MD, USA; 5 Department of Family Medicine and Karmanos Cancer Institute, Wayne State University, Detroit, MI, USA; 6 Department of Preventive Medicine and Pathology, and Norris Comprehensive Cancer Center, USC Keck School of Medicine, University of Southern California, Los Angeles, CA, USA

## Abstract

**Background:**

Exploring spatial-temporal patterns of disease incidence through cluster analysis identifies areas of significantly elevated or decreased risk, providing potential clues about disease risk factors. Little is known about the etiology of non-Hodgkin lymphoma (NHL), or the latency period that might be relevant for environmental exposures, and there are no published spatial-temporal cluster studies of NHL.

**Methods:**

We conducted a population-based case-control study of NHL in four National Cancer Institute (NCI)-Surveillance, Epidemiology, and End Results (SEER) centers: Detroit, Iowa, Los Angeles, and Seattle during 1998-2000. Using 20-year residential histories, we used generalized additive models adjusted for known risk factors to model spatially the probability that an individual had NHL and to identify clusters of elevated or decreased NHL risk. We evaluated models at five different time periods to explore the presence of clusters in a time frame of etiologic relevance.

**Results:**

The best model fit was for residential locations 20 years prior to diagnosis in Detroit, Iowa, and Los Angeles. We found statistically significant areas of elevated risk of NHL in three of the four study areas (Detroit, Iowa, and Los Angeles) at a lag time of 20 years. The two areas of significantly elevated risk in the Los Angeles study area were detected only at a time lag of 20 years. Clusters in Detroit and Iowa were detected at several time points.

**Conclusions:**

We found significant spatial clusters of NHL after allowing for disease latency and residential mobility. Our results show the importance of evaluating residential histories when studying spatial patterns of cancer.

## Background

From 1975 to 2000 in the United States, the annual age-adjusted incidence rate of non-Hodgkin lymphoma (NHL) increased more than 75% from 11.1 to 19.8 per 100,000 person-years [1]. The incidence rate has leveled recently, to 19.6 per 100,000 person-years between 2003-2007 [2]. An increase in incidence also occurred in other developed countries; for example, incidence increased across Europe since the 1960s [3]. The cause of the increases is largely undetermined and little is known in general about the etiology of NHL.

Incidence of NHL increases with age, is 40-70% higher in whites compared to blacks, and is higher in men [4]. Established risk factors include specific viruses, immune suppression, and a family history of hematolymphoproliferative cancers [5]. Other putative risk factors include specific genetic polymorphisms [6-8], certain occupations [9], and environmental exposures such as pesticides [10], polychlorinated biphenyls (PCBs) [11,12], and organic solvents [13]. Previous studies have found higher risk of NHL among persons living in areas with industrial waste exposure [14-17]. Associations with these risk factors are generally moderate-to-weak in strength or inconsistent in the literature. Taken together, the established and putative risk factors account only for a small proportion of the total annual NHL cases [5]. In addition, little is known about the latency period that might be relevant for environmental exposures. Novel approaches are needed to generate insights into the etiology of NHL.

Investigating spatial-temporal patterns of disease incidence through cluster detection analysis identifies areas with significantly different disease risks than the overall population under study. A cluster is typically considered to be an area of significantly elevated disease risk, but it could also be an area of significantly lowered risk. We note the distinction in goals between detecting local clusters and approaches to describe global clustering of disease, the general tendency for cases to occur nearer other cases than one might expect under equal risk [18-20]. The identification of local clusters can lead to the development of specific hypotheses to explain the pattern of risk and reveal important clues about disease etiology [21].

However, limitations in existing methods for evaluating clusters in space and time in epidemiologic studies have hindered cluster analyses. No previous cluster analyses of NHL has adjusted for individual-level risk factors and modeled disease latency in one unified statistical framework. Existing cluster detection techniques as applied to case-control or cohort studies [22-24] do not allow for simple adjustment of confounding variables using all data simultaneously in the cluster detection model. Typically, either a pre-processing regression analysis must be done before the cluster analysis to adjust for risk factors or several cluster analyses must be performed on strata of the data, where stratified analyses will be limited by small sample sizes. In a cluster analysis, the residential locations of study subjects at time of diagnosis are typically considered a surrogate for environmental exposures defined broadly to include lifestyle factors such as diet, in addition to pollutants, and spatially-varying socioeconomic factors. Using the diagnosis location makes the unrealistic assumption that individuals do not migrate and that the latency between causal exposures and diagnosis of disease is negligible [25]. Cluster studies that make use of residential history data in epidemiologic studies typically assume one latency period with little empirical justification. Some studies have included all historical residential locations for each subject in one statistical model with an assumption of independent observations, which can bias model results by ignoring many correlated records in the model for each subject [26-28].

To date, there have been no published spatial-temporal cluster studies of NHL using residential history data. We evaluated residential histories for NHL cases and controls in the National Cancer Institute (NCI) Surveillance, Epidemiology, and End Results (SEER) NHL (NCI-SEER NHL) case-control study to determine if there were statistically significant spatial clusters of NHL using several time windows of residential location. The NCI-SEER NHL study is a large, multi-center, population-based case-control study of NHL in four areas of the United States (Detroit, Los Angeles, Seattle, Iowa). We developed a statistical approach for cluster analysis based on the established generalized additive model (GAM) framework. We extended previous work [26-29] applying GAMs to model spatial variability in disease risk by considering the temporal aspect of disease risk as a model selection problem in a GAM. We hypothesized that after adjusting for NHL risk factors there would be significant spatial clusters of NHL cases due to unmeasured environmental risk factors, and that earlier residential locations, up to 20 years before diagnosis, would model NHL risk better than residence at diagnosis.

## Methods

### Study population

The study population, described in detail previously [5,6,30], included 1,321 NHL cases diagnosed between July 1, 1998 and June 30, 2000, aged 20 to 74 years, from four SEER registries. The four SEER areas were: Macomb, Oakland, and Wayne counties for Detroit; Los Angeles County; King and Snohomish counties for Seattle, and the state of Iowa. Self-reported HIV-positive cases were excluded from the study. Population controls (n = 1,057) were selected from residents of the four SEER areas using random digit dialing (< 65 years) or Medicare eligibility files (≥ 65 years), frequency matching to cases by age (within 5-year groups), sex, race, and SEER area. Controls with a history of NHL or known HIV infection were excluded. Among eligible subjects contacted for an interview, 76% of cases and 52% of controls participated in the study.

Interviews were conducted in 1998 to 2000. A computer-assisted personal interview was administered that contained questions about various potential risk factors for NHL, including occupation, home and garden use of pesticides, diet, hair dyes, alcohol and tobacco, and viruses [5,6,30]. All participants were sent a lifetime residential calendar in advance of the in-person interview. They were asked to provide their complete address for every home they lived in from birth to the current year, indicating the year they moved in and out by a vertical line between each home; they were also asked to provide information about temporary or summer homes where they lived for a total of two years or longer. Interviewers reviewed the residential calendar with respondents and probed to obtain missing information.

Residential addresses were matched to geographic address databases to yield geographic coordinates. Address matching was done using ArcView 3.2 software (ESRI, Inc., Redlands, California) and Geographic Data Technology's MatchMaker SDK Professional Version 4.3 street database (Geographic Data Technology, Inc., Lebanon, New Hampshire). After address matching, there were 1,166 (88%) participating cases and 943 participating controls (89%) with geographic coordinates for the residence at diagnosis (similar reference date for controls).

We excluded residences that were address matched to the level of a populated place, county centroid, or state centroid. In addition to exact matches, we included residences matched to intersections (8.8% of addresses) or ZIP Code centroids (5% of addresses). Most of the addresses matched to the ZIP Code level were located in Iowa; there was no obvious spatial pattern in these addresses. To maintain a consistent dataset while exploring the effect of different residence location time periods on the risk of NHL, we included participants who had a 20-year history of continuous residential location within one of the four SEER centers. The percent of addresses that were matched to a populated place or state or county centroid (excluded in analysis) generally increased with increasing time before enrollment, requiring a balance between potentially long latencies of interest and data quality. For each participant, we included all residential locations within 2 miles of each study area boundary. A total of 842 cases (64%) and 680 controls (64%) met our criteria for inclusion in this analysis. Summary statistics for the frequency-matching variables are listed in Table 1. The cases and controls were distributed fairly evenly across the four study centers. Residential mobility was similar across the study areas. The median number and quartiles (lower, upper) of addresses per subject in each study center were 2 (1, 2) in Detroit, Iowa, and Seattle, and 1 (1, 3) in Los Angeles.

**Table 1:** Descriptive statistics for analysis population and NCI-SEER NHL study population

	Cases (n = 842)	Controls (n = 680)	Analysis Total (n = 1522)	Study Total (n = 2378)
Study Center, %				

Detroit	25	21	24	22

Iowa	32	31	31	27

Los Angeles	23	24	23	25

Seattle	20	24	22	26

Age, mean years				

Detroit	57	61	59	56

Iowa	61	63	62	59

Los Angeles	60	60	60	56

Seattle	60	61	61	57

Males, %				

Detroit	53	45	50	52

Iowa	50	52	51	51

Los Angeles	55	52	54	55

Seattle	49	51	50	53

White, %				

Detroit	82	75	79	76

Iowa	98	99	99	98

Los Angeles	77	61	70	66

Seattle	94	93	93	90

Note: Analysis population excluded subjects address matched to the level of a populated place, county centroid, or state centroid and/or who did not live in the study area for 20 years.

### Statistical models

We used generalized additive models [31,32] to model spatially the probability that an individual had NHL within each SEER center study area. GAMs have been used to model the probability of disease in other case-control cluster analyses [26-28]. Our GAM-based approach is different from previous approaches in how we modeled multiple residence periods, treating selection of the optimal residential time period as a model selection problem. The optimal time period can be considered as the time in years before diagnosis of NHL or reference date (controls) when etiologically-relevant exposure(s) occurred and we hereafter refer to it as the lag time. We fitted crude models and models adjusted for several factors associated with NHL in our study population and available for all cases and controls in our analysis, including age, gender, race, education, and home treatment for termites before 1988 as a surrogate for exposure to the pesticide chlordane. Other NHL risk factors for this study population, such as PCB levels in the current home and levels of PCBs and furans in the blood, were available for only a subset of the subjects and were omitted in this analysis to maximize the number of subjects studied.

We considered a binary response variable *Y *for NHL case status with associated *P*(*Y *= 1) = *p*(*s,x*) depending on the explanatory variables *x *and the spatial location *s*, which consists of the coordinates (*s*_1_,*s*_2_). Given the residential locations *s*_*t *_for subjects at a particular time *t*, the log odds of being a case is modeled as(1)

where the left-hand side of the equation is the log of the disease odds for subject *i*, *α *is an intercept, *β *is a column vector of regression coefficients, X_*i *_is a row of the matrix of covariates, and *Z*_*t*_(s_*t*_) is a smooth function of the residential locations at a particular time *t*. The GAM framework is flexible, as the model in equation (1) becomes a crude spatial model when no covariates are specified, a logistic regression model when no spatial smoothing term is specified, and the null model when neither covariates nor a spatial smoothing term are specified (intercept only). Adjusting for known risk factors may explain a cluster observed in a crude analysis or may identify clusters not seen in a crude analysis. Any spatial cluster observed after adjustment for known risk factors would be the subject of further study to identify unknown spatially varying risk factors.

The function *Z*_*t*_(s) is a spatial smoothing of the locations and models spatial variation not explained by the covariates. The spatially smoothed term may be considered a surrogate for unmeasured environmental exposures at a specified time. The smoothing function is used as a measure of the density of cases relative to controls over space. This approach models cases and controls as a marked heterogeneous Poisson point process with intensity *λ*(s) = *λ*_1_(s) + *λ*_0_(s), where *λ*_1_(*s*) is the intensity of cases and *λ*_0_(*s*) is the intensity of controls. The related technique of kernel intensity estimation [33,34] uses the ratio of the intensities of point processes for controls and cases to yield a spatial odds function, but because of its limitation to easily adjust for covariates, we selected the GAM framework.

**Figure 1:**
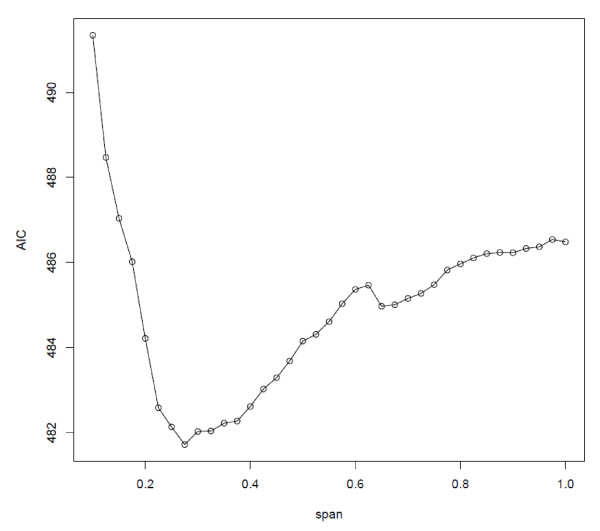
**Akaike information criterion (AIC) and span values of the crude spatial model for Los Angeles at a residential time lag of 20 years**.

We treated the spatial smooth as a bivariate smoothing function over both spatial dimensions and used loess, or locally weighted scatterplot smoothing, as the type of smoother [35,36]. This type of bivariate smoother has been used in a GAM in other case-control cluster studies [26-28]. The smoothing function has a span parameter that controls the amount of smoothing in the local odds ratios. In loess, this span is the proportion of the data that is used to estimate the function at any one particular location *s*. For example, a span of 0.5 means that 50% of the data will be used to produce the function estimate at each data point. The data used in estimating the function are the closest in space to location *s*. The span parameter must be estimated, and we evaluated the span over a large range of values to minimize the Akaike Information Criterion (AIC) [37]. We selected the smallest span among the spans associated with local minima AIC when the difference between the local minimum AIC and the global minimum AIC was not meaningful. We used the previously suggested guideline of 3 as a meaningful difference in AIC [38,39]. The approach of using smaller spans with local minimum AIC to emphasize local variation in risk has been used previously [27,28]. As an example of span selection, the selected span of 0.275 in the crude model for the Los Angeles center at a residential time lag of 20 years had the globally minimizing AIC (Figure 1). The spans selected for all latency periods for each study area are listed in Table 2. As a sensitivity analysis, we also repeated the analysis with the global span if it was different from the chosen span.

**Table 2:** Smoothing span values for the crude and adjusted models for the SEER study areas

	Years Before Diagnosis				
Study Area	0	5	10	15	20
Detroit					

crude	0.600	1.000	0.625	0.625	0.600

adjusted	0.600	1.000	0.625	0.600	0.600

Iowa					

crude	0.600	0.600	0.525	0.600	0.625

adjusted	0.600	0.575	0.525	0.600	0.625

Los Angeles					

crude	0.600	0.600	0.275	0.325	0.275

adjusted	1.000	1.000	1.000	1.000	0.275

Seattle					

crude	0.800	0.800	1.000	0.625	0.625

adjusted	0.800	0.800	1.000	1.000	1.000

We estimated the GAM model parameters in the statistical analysis software R [40] using the gam package, which is written by Trevor Hastie and is an implementation of the GAM framework of Hastie and Tibshirani [31]. While there are other options available for smoothing spline functions in gam and in the R package mgcv, we chose loess because its span parameter is the most readily interpreted.

### Mapping risk

To produce a map of local odds ratios (ORs) of NHL from the GAM model, we first estimated all parameters for the model expressed in equation (1) using the study data. We then predicted the log odds over a rectangular grid over the study area using the estimated model parameters. We used a 50 × 50 grid (2,500 cells) based on the minimum and maximum coordinates of each study area. We assigned covariate values to the grid for adjusted models. To provide an interpretable odds ratio map, we used the analysis population for each SEER center as the reference and divided the odds from the spatial model at each grid point by the odds from the null model. This approach has been used previously [28] and yields a local odds ratio that is interpretable in relation to the overall study area. For example, an OR of 1.8 at a specific location means that the rate of NHL there is elevated 80% compared with the entire study area.

To evaluate the local odds ratios for statistical significance in crude and adjusted models, we used pointwise permutation distributions. This type of assessment is common practice in kernel density estimation [18,34] and has also been used in other GAM-based approaches [26-28]. Informally, to identify areas of significantly elevated disease risk, or clusters, we determine if the observed elevated odds ratios are more extreme than we would expect under the null hypothesis that case status does not depend on location. Pointwise permutation distributions are built through iterative Monte Carlo randomization of case status to compare the observed local odds ratio at a location to the distribution of local odds ratios under the null hypothesis at that location [18]. The Monte Carlo randomization first conditions on the residential locations and then randomizes the case labels among the fixed locations. For each randomization of the case labels, the spatial model parameters are estimated and then used to predict the local odds ratios on the grid. This procedure is repeated 999 times to build a distribution of local odds ratios at each location on the grid. We identified areas of significantly elevated risk as those areas that had an observed odds ratio in the upper 2.5% of the ranked permutation distribution of odds ratios. Similarly, we identified areas of significantly lowered risk of NHL as those having an observed odds ratio in the lower 2.5% of the ranked permutation distribution. Clusters of elevated risk are identified with contours of the 97.5 percentile of the pointwise permutation distributions of local odds ratios, therefore they are significant at the 0.05 level (assuming a two-tailed distribution).

It is noteworthy that the Monte Carlo randomization technique for evaluating significance provides pointwise statistical inference, not overall inference due to the multiple locations of evaluation and the correlation in local odds ratios from the sharing of data in the smoothing function at nearby locations [19]. As a result, this method should be used to identify clusters of significantly elevated and decreased risk, but not to identify the most likely cluster in the study area.

### Evaluation of residential lag time

To determine which residential location time period was most associated with NHL risk, we included in models for each center the smoothed spatial pattern of subject residences at the time of diagnosis and also at four time periods before diagnosis. We chose 5, 10, 15, and 20 years for both the crude and adjusted models. We tested the effect of different lag times on NHL risk through analysis of deviance (ANODEV), testing for significant differences between model deviances from the model with no smoothing term and from the five models with smoothing terms (one for time at diagnosis, four with lag times). Specifically, we estimated equation (1) for time at diagnosis (*Z*_0_) and for each lag time of interest (*Z*_*k*_) and statistically tested the deviances from the models with a *Z *term and the deviance from the model with no *Z *term. The difference in deviances for two nested models approximately follows a chi-square distribution with an associated p-value. The p-value for each ANODEV test indicates the probability of achieving a reduction in deviance equal to the difference in model deviances for a number of degrees of freedom equal to the difference of model degrees of freedom when using one model nested in another model. These p-values should be interpreted with caution, as recent work has shown that chi-square p-values for smoothed terms in GAMs tend to have inflated type I error rates [41]. A significantly lower deviance from a model with a lag time of *k *years means that using the smoothed pattern of residential locations from *k *years ago significantly explains overall NHL risk. The lag-time model with the smallest p-value from analysis of deviance best explains the risk of NHL. The test of the term for smoothed residential locations tests for an overall spatial pattern in NHL risk, and as such may be considered as a type of global test of spatial variation of disease. This global test evaluates a different property of the disease pattern than the Monte Carlo randomization process described earlier, which tests for local clusters of elevated NHL risk.

### Evaluation of potential selection bias

Because response rates were relatively low for both cases and controls, we investigated the possibility that selection bias affected the results of our spatial cluster analysis. The concern was that clusters detected in study participants could be due to differential participation among cases and controls. For example, a high density of nonparticipating controls and low density of nonparticipating cases in an area could produce an artificial cluster of elevated risk. To explore this possibility, we performed additional cluster analyses among study participants and nonparticipants in each center using the GAM approach described above, but limited to a single time point.

Current addresses and demographics (age, race, gender) were available for all eligible cases (from the registry) and for controls 65 and older (from Centers for Medicare & Medicaid Services). Eligibility of younger controls was determined by a telephone survey in which gender, age, and the residential address were obtained. We first performed a spatial cluster analysis for participants only with crude models and models adjusted for only age and gender. We then performed a cluster analysis for participants and nonparticipants together. We were primarily interested in clusters of elevated risk in participants that were not present in the analysis with participants and nonparticipants.

Because nonresponse to the telephone survey resulted in incomplete ascertainment of eligible controls less than 65 years of age, we also performed a separate evaluation of nonresponse bias restricted to participants aged 65 years or more.

## Results

In our analysis of residential locations at several time periods, the most significant lag time was 20 years according to both crude and adjusted models (Table 3) in three centers: Detroit (crude and adjusted models p-value = 0.07), Iowa (crude model p-value = 0.21, adjusted model p-value = 0.14), and Los Angeles (crude model p-value = 0.003, adjusted model p-value = 0.03). In Seattle, the most significant lag time was 10 years for both the crude (p-value = 0.20) and the adjusted (p-value = 0.15) models. Overall, the results showed that a residential lag time of 20 years best explained risk of NHL associated with spatially-dependent exposures in our study.

**Table 3:** Analysis of deviance test p-values for crude and adjusted models in the SEER study areas

	Years Before Diagnosis				
Study Area	0	5	10	15	20
Detroit					

crude	0.116	0.179	0.129	0.099	0.071

adjusted	0.091	0.118	0.075	0.089	0.072

Iowa					

crude	0.302	0.277	0.244	0.264	0.211

adjusted	0.212	0.178	0.175	0.155	0.144

Los Angeles					

crude	0.098	0.115	0.019	0.032	0.003

adjusted	0.722	0.698	0.410	0.283	0.029

Seattle					

crude	0.477	0.295	0.203	0.270	0.684

adjusted	0.534	0.289	0.147	0.318	0.829

Among the adjusted models, only the lag time of 20 years for Los Angeles was statistically significant at the 0.05 level. The lag time of 20 years for Detroit was marginally statistically significant (p-value=0.07). It is worth noting that the p-value for a lag time of 20 years for Los Angeles was an order of magnitude lower than for other lag times in this study center. Such a marked difference among lag times was not found in the other study centers. It is also noteworthy that whereas p-values for the analysis of deviance of crude models in Los Angeles for the lag times of 10, 15, and 20 years were statistically significant at the 0.05 level, after adjusting for known risk factors the contribution of the residual spatial term to the log odds of NHL was no longer statistically significant for lag times of 10 and 15 years.

**Figure 2:**
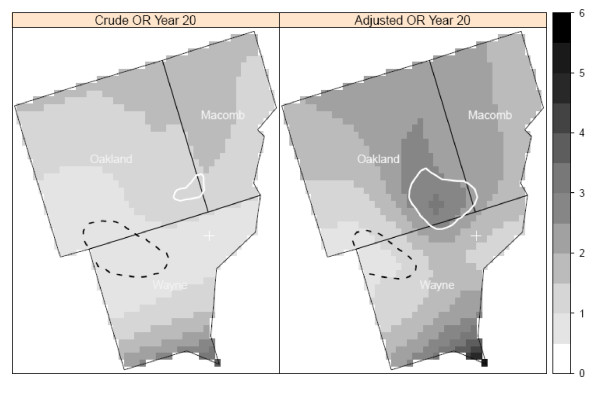
**Crude and adjusted local odds ratios (OR, scale at right) for NHL at a residential lag time of 20 years in the Detroit study area**. Clusters of statistically significant elevated odds ratios are identified with a solid white line and statistically significant lowered odds ratios are identified with a dashed black line. Crude model: span = 0.6 (p-value = 0.07); Adjusted model: span = 0.6 (p-value = 0.07). Model adjusted for age, gender, race, education, and home termite treatment before 1988.

Using the most significant lag time identified in crude and adjusted models, we found significant clusters of elevated NHL risk in three of the study centers (Detroit, Iowa, and Los Angeles). In the Detroit center, there was an area of elevated risk in southeast Oakland County near the junction of Oakland, Macomb, and Wayne counties at a lag time of 20 years (Figure 2). In addition to the area of significantly elevated risk, we also found a cluster of relatively low risk of NHL in northwestern Wayne County. The presence and general location of clusters were consistent between the crude and adjusted models; however, there were differences in the shapes of the clusters. In Detroit, the cluster detected by the adjusted model is larger and has a greater risk of NHL than does the cluster in the same area detected by the crude model.

**Figure 3:**
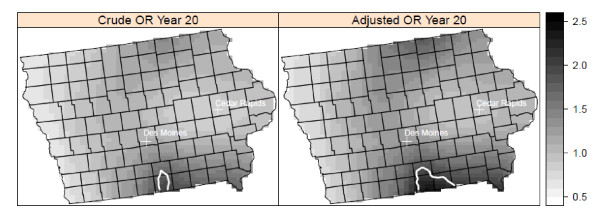
**Crude and adjusted local odds ratios (OR, scale at right) for NHL at a residential lag time of 20 years in Iowa**. Clusters of statistically significant elevated odds ratios are identified with a solid white line and clusters of statistically significant lowered odds ratios are identified with a dashed black line. Crude model: span = 0.625 (p-value = 0.21); Adjusted model: span = 0.625 (p-value = 0.14). Model adjusted for age, gender, race, education, and home termite treatment before 1988.

**Figure 4:**
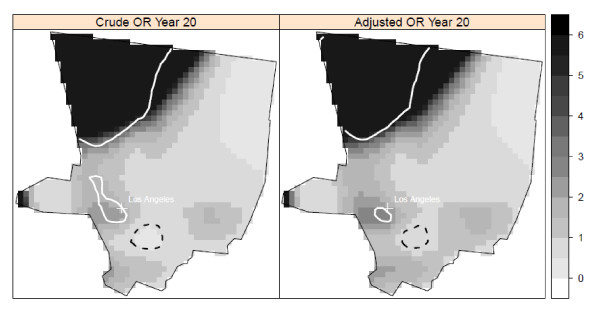
**Crude and adjusted local odds ratios (OR, scale at right) for NHL at a residential lag time of 20 years in the Los Angeles study area**. Clusters of statistically significant elevated odds ratios are identified with a solid white line and statistically significant lowered ORs are identified with a dashed black line. Crude model: span = 0.275 (p-value = 0.003); Adjusted model: span = 0.275 (p-value = 0.03). Model adjusted for age, gender, race, education, and home termite treatment before 1988.

**Figure 5:**
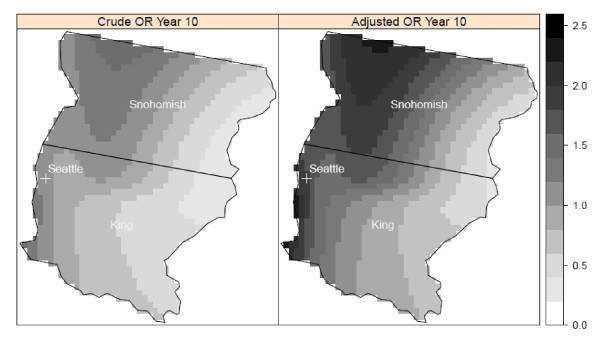
**Crude and adjusted local odds ratios (OR, scale on right) for NHL at a residential lag time of 10 years in the Seattle study area**. Crude model: span = 1 (p-value = 0.20); Adjusted model: span = 1 (p-value = 0.15). Model adjusted for age, gender, race, education, and home termite treatment before 1988.

There was a statistically significant cluster of elevated risk in south-central Iowa at a lag time of 20 years that included most of Wayne, and parts of Appanoose, Davis, and Lucas counties in the adjusted model (Figure 3). There were no clusters of lowered risk. There were two significant areas of elevated risk in the Los Angeles study area at a 20-year lag time (Figure 4). One cluster was located in northwestern Los Angeles County, with cases located in the large geographic but sparsely populated northwestern part of Los Angeles County. A small cluster of elevated risk was also found in the city of Los Angeles, in West Hollywood. This cluster appeared as a high risk area for NHL, corresponding to an area with a high prevalence of HIV positive individuals in an earlier study conducted at the census tract level [42]. There was also a small cluster of lowered risk detected in mostly Hispanic southeast Los Angeles County. We found no statistically significant clusters in the Seattle center at the 10-year lag time in either the crude or adjusted models (Figure 5). Our sensitivity analysis of selected span values revealed that the patterns of local odds ratios were smoother with the larger spans, but the locations of clusters generally remained the same (data not shown).

**Figure 6:**
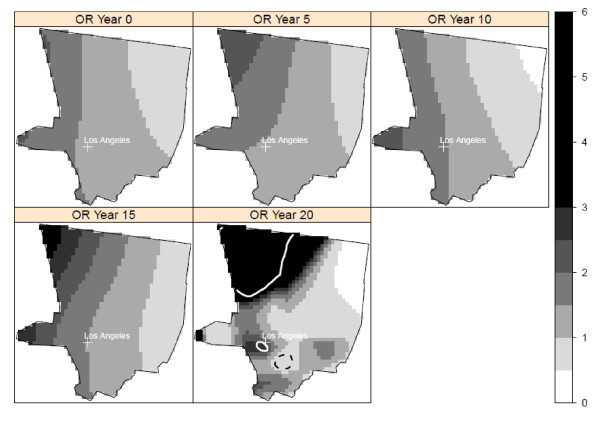
**Adjusted local odds ratios for NHL at time of diagnosis and four residential lag times in Los Angeles**. Clusters of statistically significant elevated odds ratios are identified with a solid white line and statistically significant lowered odds ratios are identified with a dashed black line. The optimal span size was used for each residential lag time. Models adjusted for age, gender, race, education, and home termite treatment before 1988.

While our primary approach was to evaluate spatial clusters at the lag time most strongly associated with risk of NHL, we also plotted the local odds ratios and significant contour lines at the other time periods to determine if the local clusters were detected at multiple time lags. We used the optimal span for each lag time. In Detroit, the area of elevated risk was present at the time of diagnosis and at 5 and 15 years before diagnosis, in addition to 20 years. The area of elevated risk in Iowa was detected at all five time periods. In contrast, the clusters of elevated and decreased risk in Los Angeles were found only at the 20-year lag time (Figure 6). No clusters were found in Seattle at any time periods.

**Figure 7:**
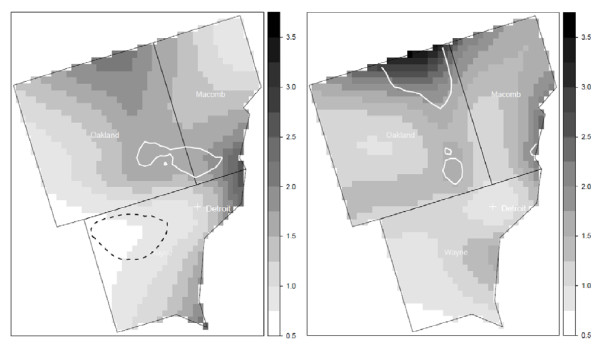
**Adjusted local odds ratios for NHL for study participants (left) and all eligible cases and controls (right) in Detroit at the time of study enrollment**. Clusters of statistically significant elevated odds ratios are identified with a solid white line and statistically significant lowered odds ratios are identified with a dashed black line. Participants: span = 0.6 (p-value = 0.05); All eligible: span = 0.325 (p-value = 0.08). Models adjusted for age and gender.

**Figure 8:**
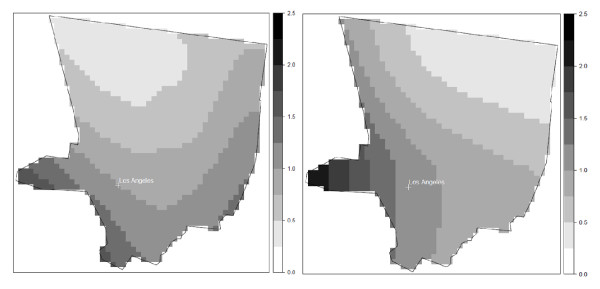
**Adjusted local odds ratios for NHL for study participants aged 65 years or more (left) and eligible cases and controls aged 65 years or more (right) in Los Angeles at the time of study enrollment**. Participants: span = 1 (p-value = 0.62); Eligible: span = 1 (p-value = 0.16). Models adjusted for age and gender.

The results from the cluster analyses for all participants versus all eligible cases and controls were generally similar for each study area. For example, the pattern of elevated risk in Detroit at time of diagnosis for all eligible cases and controls was similar to that for participants, although the location of statistically significant clusters shifted somewhat (Figure 7). The cluster of lowered risk in Wayne County was not observed when including all eligible cases and controls. However, the area of significant elevated risk found in participants of the study is not fully explained by selection bias, as much of the cluster in southern Oakland County remains after including nonparticipants in the analysis. The results from the cluster analyses for participants aged 65 or more and eligible cases and controls aged 65 years or more were also similar across the study areas. For example, the overall pattern of risk of NHL in Los Angeles at time of diagnosis is similar with no areas of statistically significant risk (Figure 8).

## Discussion

Our approach to modeling spatial variation in disease risk extends earlier work with generalized additive models by evaluating several residential time windows as a model selection problem using residential histories in a case-control study. The spatial analysis of NHL risk in the NCI-SEER NHL study provided evidence that residential location at 20 years, the longest lag time we evaluated, explained NHL risk better than residence location at times closer to diagnosis. We found that there were areas of statistically significant risk of NHL in several of the study areas. Detroit and Iowa each had a cluster of elevated risk after adjusting for known individual-level risk factors. The cluster in Iowa was found in five residential time periods and the cluster in Detroit was found in four time periods. The Los Angeles study area had two clusters that were present only at a lag time of 20 years. The Seattle study area had no clusters. Despite the presence of local clusters, there was not consistent evidence across the study areas for significant overall spatial variation in NHL risk. It is possible to have local clusters without an overall pattern of clustering [18,19,43,44]. Our results demonstrate the importance of considering past residential locations in a cluster analysis. Here, we elaborate on some features of our analysis, including the evaluation of various lag times, adjustment for risk factors, and some specific considerations of the modeling approach.

To our knowledge, this is the first spatial cluster analyses of a case-control study of NHL using residential histories to account for residential mobility. Cluster studies often use the residence location at time of diagnosis, thereby assuming the relevant environmental exposure or risk factor occurred at the diagnosis address. This will be true for some subjects, but certainly not for others. Previous cluster studies [26-28] using generalized additive models included in one model all possible residences for each subject before a lag time of interest, resulting in a biased model with several correlated records per subject. With such an approach, a detected cluster could be the result of a few cases moving around within a small area over time [27]. A previous study restricted one analysis to the residence of longest duration and found somewhat different results in cancer cluster locations than when using multiple addresses per subject with a latency of 20 years [27]. Previous studies have also assumed certain lag times without quantitative justification of the particular choice. In contrast, we treated the selection of the lag time as a model selection problem, where the smoothed functions of the residences at specific lag times are treated as model terms; the term that best explains disease risk suggests the most relevant lag time. While our modeling approach allows for testing the significance of different time periods, the model assumes one relevant residential lag time, i.e. one location for exposure. It is possible, however, that exposures occur at more than one residential location. In other words, exposure could be cumulative over time and space. To consider this, in future work we will develop methods to include several residential locations for each person in one model. Such an approach is a step toward encompassing life-course environmental exposures, the idea of the "exposome" [45].

In our study, we adjusted for several potential confounding variables in a spatial cluster model. Often cluster studies do not adjust for known risk factors, aside from basic demographics. Any covariate that has a spatial pattern and is associated with the outcome could cause confounding of the association of the outcome and unmeasured environmental exposure, as represented by the smoothed function of residential locations. After adjustment for confounders, an observed cluster may become nonsignificant. Conversely, adjusting for known risk factors can also identify hidden clusters in a crude analysis. Other cluster detection techniques, such as the local scanning method in SaTScan [22,23] and Q-statistics [24], do not allow for adjustment of confounding variables in a case-control or cohort study in a unified statistical cluster-analysis model. In our study, adjusting for known risk factors made certain clusters more prominent and revealed a pattern of increased risk for NHL in Detroit and Iowa. A cluster in Los Angeles decreased in size with adjustment. While we adjusted for several risk factors, our adjustment did not include all possible risk factors of NHL. In future work, we will explore the observed clusters for the presence of significant variables in other datasets, such as the U.S. Census, Environmental Protection Agency (EPA) Toxic Release Inventory, and the Census of Manufacturers and the Census of Agriculture.

There are several aspects of the generalized additive model framework to consider when performing cluster analysis, including the span selection and possible edge effects. Span selection is important because patterns in the local odds ratio maps derived from GAMs depend on the span. A small span will reveal more local variation in risk, while a larger span will produce a smoother pattern of risk. Using an automatic search routine to select the span with the smallest Akaike information criterion may lead to selecting a span with a local minimum AIC and not a global minimum. To avoid this, we selected the span visually from a graph of AIC values for a range of spans. However, this approach can suggest several span values with local minima that are very close in AIC to the global minimum. We favored smaller values of the span when the difference in AIC for a local minimum and a global minimum was not meaningful to identify important features of the disease point process. Sensitivity analysis with larger spans did not change the presence and locations of observed clusters.

Generalized additive models with spatial smoothing terms, and kernel density estimation more generally, are susceptible to edge effects, which can result in biased estimates of risk on the periphery of a study area. To minimize edge effects in this approach, we used a loess function for smoothing over residential locations. Loess uses a tri-cube weight function that gives less weight to data points farther from the estimation point [31,35,36]. Due to this weighting function, loess should exhibit smaller edge effects than an alternative smoother based on nearest neighbors with equal weights [28]. In a previous simulation study, Webster et al. [28] found little evidence of edge effects interfering with estimating the true odds ratio surface using loess as the spatial smoother in a generalized additive model. We also sought to minimize edge effects to some degree by including residential locations within 2 miles of each study area boundary.

In general, results of a cluster analysis should be interpreted carefully due to potential selection bias. Response rates were relatively low for both cases and controls in the NCI-SEER NHL study. Differences in the distributions of respondents and nonrespondents may make case and control groups unrepresentative of the base population in terms of exposure prevalence. However, the risk estimate will only be biased if a risk factor differentially influences participation among controls and cases [46]. Previously, we analyzed spatial patterns in nonresponse separately by study center and by case status using a spatial scan statistic [30]. Two significant elliptical clusters in Detroit and Los Angeles were nonsignificant after adjusting for demographic factors. We also found that the nonresponse bias in NHL risk associated with education level was not large. De Roos et al. [47] found evidence of selection bias in the NCI-SEER study when investigating proximity to industrial facilities and risk of NHL. Current residences of participants were less likely to be located within 2 miles of an industrial facility than were those of nonparticipants. The differences in proximity to industry by participation were accounted for by variation in select census block group-level demographic variables. Although proportions of participants and nonparticipants living within 2 miles of an industrial facility were significantly different, the association between proximity of current residence to one or more industry and NHL risk did not differ by participation. In our comparative cluster analyses of participants and all eligible cases and controls at time of study enrollment, we did not find substantial differences in patterns of elevated risk. Clusters of elevated risk in participants were not explained by including nonparticipants in the analysis. The results suggested that the response bias was not responsible for the clusters of elevated risk detected among study participants. Our assessment of bias, however, was limited to only residences at time of selection into the study.

## Conclusions

After adjusting for several known risk factors, we found evidence of several clusters of elevated NHL risk in the NCI-SEER NHL study. We also found that long lag times of residential location were more likely than shorter lag times to result in significant clusters of elevated NHL risk in several study areas. Results of this study will lead to future investigations to evaluate possible reasons for the significant clusters, which may lead to new hypotheses about the etiology of NHL. We performed our cluster analyses using generalized additive models, which provide a unified statistical framework for estimating disease risk spatially and assessing significance of elevated risk for case-control and cohort data. It is straightforward to adjust for covariates and evaluate several temporal lags in this statistical framework. This study serves as an illustrative example for those interested in performing space-time cluster analysis of diseases without well-known etiologies and this approach can be useful in the generation of new hypotheses.

## List of Abbreviations

OR: Odds Ratio; NHL: Non-Hodgkin Lymphoma; EPA: Environmental Protection Agency; ANODEV: Analysis of Deviance; AIC: Akaike Information Criterion; PCB: Polychlorinated Biphenyl; NCI: National Cancer Institute; SEER: Surveillance, Epidemiology, and End Results; GAM: Generalized Additive Model

## Competing interests

The authors declare that they have no competing interests.

## Authors' contributions

DW conceived of the study, performed the statistical analysis, and drafted the manuscript. MW participated in the design of the study and helped to draft the manuscript. ADR, JC, RS, WC, and LM participated in the design and conduct of the case-control study. All authors read and approved the final manuscript.
